# The Effect of Salpingectomy on Ovarian Reserve Using Two Different Electrosurgical Instruments: Ultrasonic Shears Versus Bipolar Electrocautery

**DOI:** 10.7759/cureus.59434

**Published:** 2024-05-01

**Authors:** Rinchen Zangmo, Gayatri Suresh, Avir Sarkar, Sivalakshmi Ramu, K K Roy, Kaloni Subramani, Priyanka Das

**Affiliations:** 1 Obstetrics and Gynaecology, Luton and Dunstable Hospital United Kingdom, Bedfordshire, GBR; 2 Obstetrics and Gynaecology, All India Institute of Medical Sciences, Rishikesh, Rishikesh, IND; 3 Obstetrics and Gynaecology, Employees' State Insurance Corporation (ESIC) Medical College and Hospital, Faridabad, Faridabad, IND; 4 Obstetrics and Gynaecology, All India Institute of Medical Sciences, New Delhi, New Delhi, IND

**Keywords:** in vitro fertilization (ivf), anti-müllerian hormone, bipolar electrocautery, ultrasonic shears, follicle-stimulating hormone, operative laparoscopy, antral follicle count, ovarian reserve, total salpingectomy

## Abstract

Background

Salpingectomy is a common surgical procedure in gynecology performed for various indications. Given its proximity to the ovaries and shared vascular supply, concerns have arisen regarding compromised ovarian reserve post-salpingectomy.

Objective

We aim to study the effect of two different energy sources (group 1: salpingectomy using bipolar electrocautery followed by division with scissors versus group 2: salpingectomy with ultrasonic shears) on residual ovarian reserve. The effect on ovarian reserve was assessed using serum levels of follicle-stimulating hormone (FSH), anti-Müllerian hormone (AMH), antral follicle count (AFC), and ovarian size pre- and postoperatively.

Materials and methods

According to the inclusion criteria, 68 women were included in the study and randomized into the bipolar electrocautery group and the ultrasonic shear group. The final analysis included 34 women in the bipolar electrocautery group and 32 in the ultrasonic shear group. Preoperatively, the ovarian reserve of all women was assessed using FSH, AMH, AFC, and ovarian size. These parameters were then reassessed at three months postoperatively, and the percentage change was analyzed.

Results

The mean baseline serum AMH and serum FSH values in the bipolar electrocautery group were 3.9 ± 2.9 ng/ml and 6.6 ± 2.1 IU/L, respectively, comparable with the values in the ultrasonic shear group, where serum AMH was 3.2 ± 2.9 ng/ml and serum FSH was 7.3 ± 3.9 IU/L. AFC and ovarian size were comparable between the two groups preoperatively (bipolar electrocautery group AFC was 8 ± 2.2, ovarian size on the right side was 3.3 ± 0.7 cm and on the left was 3.2 ± 0.6 cm; ultrasonic shear group AFC was 5.7 ± 2.3, ovarian size on the right side was 3.4 ± 0.8 cm and on the left was 3.2 ± 0.8 cm). After three months of postoperative analysis, AFC showed a significant fall from the preoperative value in the bipolar electrocautery group compared to the ultrasonic shear group (AFC reduced from 8 ± 2.2 to 5.5 ± 2.3 vs. 8.6 ± 0.5 to 7.9 ± 2.3; p=0.002). The other parameters showed no statistically significant change.

Conclusion

Our study suggests that ultrasonic shear is safer than bipolar electrocautery for preserving ovarian reserve after salpingectomy. However, further research is needed to confirm these findings.

## Introduction

Salpingectomy is a commonly performed gynecological procedure for a wide variety of indications across women of the reproductive age group. Salpingectomy may be performed in cases of tubal pregnancy, pretreatment for in vitro fertilization (IVF) patients with gross hydrosalpinx, as an option for permanent sterilization, and prophylactic salpingectomy [1-3]. Opportunistic salpingectomy is aimed at the primary prevention of epithelial ovarian cancer and has been a topic of debate in the last decade. The American College of Obstetricians and Gynecologists backs opportunistic salpingectomy in high-risk groups as a primary measure of cancer prevention [2]. Laparoscopic approaches, including mini-laparoscopic modifications, are favored options for salpingectomy for their advantages of lesser tissue damage and intraoperative blood loss, quicker postoperative stay, and better cosmesis [4]. Despite the many advantages offered by the procedure, the possibility of compromising ovarian function remains an unsettled concern. Ovarian tissue compromise could stem from damage to collaterals from ovarian vessels, direct tissue injury, or lateral dissipation of energy from energy sources [3-6]. Apart from fertility concerns, a decline in ovarian function could have detrimental effects on cardiovascular health, bone strength, and even the cognitive functions of women [2]. Adopting a surgical approach with minimum ovarian damage thus becomes imperative while dealing with women, particularly in the early reproductive years. In this context, our study was designed to evaluate the effect of surgical approaches using two different energy sources for salpingectomy and their effect on residual ovarian reserve.

The study aimed to assess and compare the effect of laparoscopic salpingectomy by two different energy sources, namely bipolar electrocautery and ultrasonic shears, on ovarian reserve. Our objective was to analyze the change in serum anti-Müllerian hormone (AMH), serum follicle-stimulating hormone (FSH), antral follicle count (AFC), and ovarian size pre- and post-salpingectomy in group 1 (salpingectomy using bipolar electrocautery) and group 2 (salpingectomy using ultrasonic shear).

## Materials and methods

Study group and duration

Women attending the gynecology outpatient department at the Department of Obstetrics and Gynecology, All India Institute of Medical Sciences, New Delhi, were enrolled in the study. The Institutional Ethics Committee of All India Institute of Medical Sciences, New Delhi, gave approval prior to the commencement of the study (approval number: 896/03.01.2020). The study was carried out between March 2020 and March 2021. All participants who met the inclusion criteria were enrolled in the study.

Patients were recruited by the inclusion criteria as shown in Table 1. Preoperatively, serum AMH and serum FSH were done on the second day of the menstrual cycle, and AFC and ovarian size were also performed on the second day of the menstrual cycle for baseline data. Patients were enrolled in the study after giving written informed consent. The exclusion criteria are depicted in Table 2. Patients recruited were randomized into two groups using a computer-generated randomization program, Epi Info version 4.0 (Centers for Disease Control and Prevention, Atlanta, Georgia). Patients randomized to group 1 underwent salpingectomy using bipolar electrocautery. A bipolar energy source was applied to the mesosalpinx as close to the fallopian tube as possible, followed by division using laparoscopic scissors. In group 2, ultrasonic shear was used to coagulate and separate the tubes. Postoperatively, at three-month follow-up visits, serum AMH, serum FSH levels, AFC, and ovarian size were repeated on the second day of the menstrual cycle uniformly for all patients, and a percentage change was calculated from the preoperative values.

**Table 1:** Inclusion criteria IVF: in vitro fertilization

Inclusion criteria	
1	Any patient planned for salpingectomy with gross hydrosalpinx before the IVF procedure, permanent sterilization, and prophylactic salpingectomy
2	Age between 18 and 40 years
3	Willingness to participate in the study

**Table 2:** Exclusion criteria

Exclusion criteria	
1	Women with ovarian pathology
2	Any contraindication for laparoscopic surgery

Study design

It was a prospective randomized trial. Our study was a pilot study with no other similar previous studies. Therefore, a prior sample size calculation was not possible. A convenient sample size of at least 60 participants was considered in the ethics committee meeting, and approval was obtained for the same.

## Results

**Figure 1:**
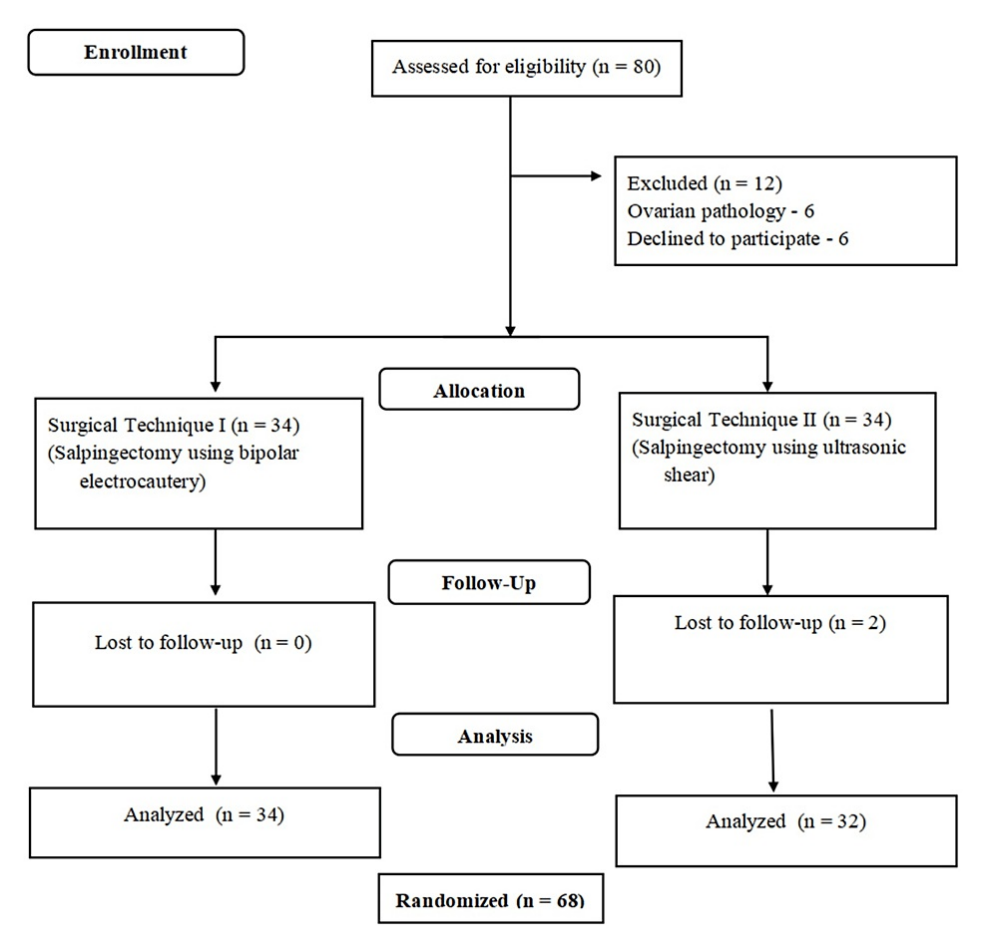
Consort diagram of the randomized controlled trial

A total of 68 patients were recruited and randomized in the study; 34 patients were randomized to the bipolar electrocautery group, and 34 patients were randomized to the ultrasonic shear (harmonic scalpel) group. Two participants from the ultrasonic shear group were lost to follow-up and were excluded from the final analysis. Figure 1 shows the consort diagram of the randomized controlled trial.

The mean age of the participants in group 1 was 34.2 ± 2.4 years and that in group 2 was 34.6 ± 2.6 years with no significant difference between the two groups. In the bipolar electrocautery group, 52.9% (n=18) suffered from secondary infertility, whereas 53.1% (n=17) patients had secondary infertility in the ultrasonic shear group (p=0.98) while the remaining patients suffered from primary infertility. The mean duration of infertility in the patients in the bipolar electrocautery group was 4.8 ± 1.9 years (n=34), whereas it was 4.9 ± 1.9 years (n=32) in the ultrasonic shear group. Hydrosalpinx and tubal factors contributed to 76.5% of cases of infertility in the bipolar electrocautery group, while these were implicating factors in 90.6% of patients belonging to the ultrasonic shear group (p=0.18). Details of baseline characteristics are summarized in Table 3.

**Table 3:** Baseline characteristics between the bipolar electrocautery and ultrasonic shear groups

	Bipolar electrocautery group (n=34)	Ultrasonic shear group (n=32)	p-value
Primary infertility patients	16 (47.1%)	15 (46.9%)	0.002
Secondary infertility	18 (52.9%)	17 (53.1%)	
Duration of infertility	4.8 ± 1.9 years	4.9 ± 1.9 years	0.58
Factors contributing infertility			
Tubal	26 (76.5%)	29 (90.6%)	0.18
Mixed	8 (23.5%)	3 (9.4%)	

On preoperative analysis, the mean serum FSH values were 6.6 ± 2.1 IU/L and 6.6 ± 0.4 IU/L in the bipolar electrocautery and ultrasonic shear groups, respectively (p=0.48). The mean serum AMH values were 3.9 ± 2.9 ng/mL in the bipolar electrocautery group and 3.8 ± 2.6 ng/mL in the ultrasonic shear group (p=0.31). With regard to the AFC preoperatively, the mean value was 8 ± 2.2 in the bipolar electrocautery group compared to 8.6 ± 0.5 in the ultrasonic shear group (p=0.17). Bilateral ovarian size was also comparable between both groups.

Intraoperatively, the mean size of hydrosalpinx noted between the bipolar electrocautery and ultrasonic shear groups was 2.9 ± 2.8 cm and 2.9 ± 2.7 cm, respectively, on the left side (p=0.98) and 2.6 ± 3.1 cm and 2.9 ± 2.5 cm, respectively, on the right side (p=0.45), showing no statistically significant difference. With regard to the laterality of hydrosalpinx, 76.6% of patients in the bipolar electrocautery group had bilateral hydrosalpinx, and 75.0% of patients had bilateral hydrosalpinx in the ultrasonic shear group (p=0.88).

The mean duration of surgery was comparable between the two groups, with a mean value of 31 ± 5 minutes in the bipolar electrocautery group and 30.3 ± 5.8 minutes in the ultrasonic shear group (p=0.70). Table 4 shows the summary of the intraoperative parameters. On analyzing the ovarian reserve parameters post-surgically, no statistical significance was noted in the serum FSH, serum AMH levels, or ovarian size. The mean post-surgery values of serum FSH were 7.3 ± 2.9 IU/L and 6.9 ± 0.4 IU/L in the bipolar electrocautery group and ultrasonic shear group, respectively (p=0.74). The mean values of serum AMH were 3.9 ± 2.9 ng/ml and 3.6 ± 2.5 ng/ml in the bipolar electrocautery and ultrasonic shear groups, respectively (p=0.47). With regards to ovarian size, it was 2.6 ± 3.1 cm in the bipolar electrocautery group and 2.9 ± 2.7 cm in the ultrasonic shear group (p=0.98). Post-surgery AFC showed a significant decline from preoperative numbers in the bipolar electrocautery group compared to the ultrasonic shear group. The values declined to a mean of 5.7 ± 2.3 in the bipolar electrocautery group and 7.9 ± 2.7 in the ultrasonic shear group (p=0.0002). No surgical complications were encountered in either of the groups intraoperatively or postoperatively. A comparison of ovarian reserve parameters between the two groups pre- and postoperatively after three months of surgery is given in Table 5.

**Table 4:** Intraoperative parameters

	Bipolar electrocautery group (n=34)	Ultrasonic shear group (n=32)	p-value
Presence of hydrosalpinx			
Unilateral	26 (76.5%)	24 (75%)	0.80
Bilateral	8 (23.5%)	8 (25%)	
Size of hydrosalpinx (cm)			
Right Side	2.9 ± 3.0	2.9 ± 2.5	0.10
Left side	3.1 ± 2.7	2.9 ± 2.7	0.65
Duration of surgery (min)	31 ± 5	30.3 ± 5.8	0.70

**Table 5:** Change in serum FSH, serum AMH, and AFC between the bipolar electrocautery and ultrasonic shear groups pre- and postoperatively FSH: follicle-stimulating hormone, AMH: anti-Müllerian hormone, AFC: antral follicle count

	Pre-surgery			Post-surgery		
	Bipolar electrocautery group (n=34)	Ultrasonic shear group (n=32)	p-value	Bipolar electrocautery group (n=34)	Ultrasonic shear group (n=32)	p-value
Serum FSH (IU/L)	6.6 ± 2.1	6.6 ± 0.4	0.48	7.3 ± 2.9	6.9 ± 0.4	0.74
Serum AMH (ng/ml)	3.9 ± 2.9	3.8 ± 2.6	0.31	3.2 ± 2.9	3.6 ± 2.5	0.47
AFC	8 ± 2.2	8.6 ± 0.5	0.17	5.7 ± 2.3	7.9 ± 2.7	0.001
Ovarian size in right (cm)	3.3 ± 0.7	3.2 ± 0.5	0.55	3.4 ± 0.8	3.3 ± 0.6	0.69
Ovarian size in left (cm)	3.2 ± 0.6	3.2 ± 0.5	0.47	3.2 ± 0.8	3.3 ± 0.6	0.39

## Discussion

Fallopian tubes are uterine appendages that derive their blood supply from the uterine and ovarian vessels. Salpingectomy therefore runs the risk of severing these collaterals from the ovaries. This, combined with possible thermal injury from the use of energy sources during salpingectomy, poses a threat to ovarian function post-surgery [7]. With the increasing incidence of salpingectomy surgeries, particularly in young women for indications like tubal ectopic pregnancy, hydrosalpinx, and prophylactic salpingectomy against epithelial ovarian malignancies, any compromise to residual ovarian function raises concern. Diminished fertility and early menopause form the major complications resulting from compromised ovarian reserve. It hence becomes imperative to adopt measures to minimize injury to the ovarian tissue during salpingectomy [7]. Several studies in the past have attempted to establish the relationship between salpingectomy and residual ovarian function. Biomarkers such as FSH, AMH, AFC, ovarian size, and response to ovarian stimulation in IVF cycles were used as surrogate markers to assess ovarian reserve in these studies [3,4,8,9].

The majority of the studies did not show any significant change in ovarian function post-salpingectomy on short-term follow-up. A randomized study by Findley et al. (2012) studied the effect of salpingectomy using monopolar and bipolar electrocautery during laparoscopic hysterectomy on ovarian reserve by measuring serum AMH at four to six weeks and three months postoperatively. No significant temporal change was noted in the serum AMH values from baseline [6]. Similarly, Morelli et al. (2013) retrospectively analyzed ovarian function in women who underwent salpingectomy with ovarian preservation along with hysterectomy compared to those without salpingectomy. AMH, FSH, ovarian size, ovarian vessel dopplers, and AFC were compared between the groups, and no significant change was noted [10]. A meta-analysis by Mohamed et al. analyzed eight studies between 2000 and 2016 in two groups. One group compared serum AMH before and after salpingectomy, whereas the other compared the alteration in serum AMH between the salpingectomy and control group. Both analyses showed no statistically significant change in the serum marker [8]. A systematic analysis conducted by Gupta et al. showed that salpingectomy done at the time of hysterectomy for benign indications with preservation of ovaries did not have any short-term adverse effects on ovarian reserve and function. Serum AMH and FSH have insignificantly decreased both preoperatively and postoperatively after three months, and the difference between both groups was also not statistically significant [11].

References on the effect of salpingectomy causing reduced ovarian reserve, prophylactic salpingectomy on reduced ovarian reserve, salpingectomy effects on IVF, and salpingectomy for ectopic pregnancy on ovarian reserve are discussed in Tables 6-9, respectively.

**Table 6:** The effect of salpingectomy showing a reduction in ovarian reserve AMH: anti-Müllerian hormone, FSH: follicle-stimulating hormone, AFC: antral follicle count, TAH: total abdominal hysterectomy, TLH: total laparoscopic hysterectomy

Author	Study type	Sample size	Type of surgery	Conclusion
Qin et al., 2016 [1]	Systematic review	13 studies	Effect of salpingectomy on ovarian reserve and ovarian function	No short-term compromise was noted in the salpingectomy group, a long-term impairment in ovarian reserve might be possible, as reflected by a decline in serum AMH
Ye et al., 2015 [12]	Retrospective study	198 unilateral salpingectomy, 41 bilateral salpingectomy, 74 no tubal surgery	Effect of salpingectomy on ovarian reserve	Salpingectomy limb had significantly lower AMH values and higher early follicular phase FSH levels compared to the women with intact adnexa
Kobayashi et al., 2022 [13]	Systematic review and meta-analysis	21-review 16-meta analysis	Salpingectomy (either unilateral or bilateral)	Salpingectomized patients (either unilateral or bilateral) have a decreased ovarian reserve
Grynnerup et al., 2013 [14]	Cross-sectional study	71	Effect of salpingectomy on ovarian reserve	Serum AMH levels were lower in salpingectomized women compared with women with tubal factor infertility and preserved fallopian tubes
Yuan et al., 2013 [15]	Prospective longitudinal study	84	Effect of hysterectomy with bilateral salpingectomy on ovarian reserve	Hysterectomy with bilateral salpingectomy compromised ovarian reserve, with the damage being most severe among younger patients
Wu et al., 2020 [16]	Meta-analysis	648 patients were included in 5 studies	Salpingectomy versus proximal tubal occlusion	Salpingectomy did more harm to ovarian reserve than proximal tubal occlusion in the short-term and long-term effects were uncertain
Atilgan et al., 2020 [17]	Experimental study	21 total, sham group, 7 proximal tubal occlusion, 7 bilateral salpingectomy	Bilateral proximal tubal occlusion and bilateral total salpingectomy by using bipolar electrocautery	Bilateral total salpingectomy in rats leads to significant damage in ovarian histopathology and the cholinergic system
Gelbaya et al., 2006 [18]	Retrospective study	168 salpingectomy proximal tubal occlusion, 103 control	Salpingectomy versus proximal tubal division	Significantly lower AFC and fewer oocytes were retrieved in the salpingectomy group compared to the group with proximal tubal division
Ni et al., 2013 [19]	Prospective cohort study	134	Unilateral, bilateral salpingectomy and tubal ligation, compared with a control group with tubal factor infertility without hydrosalpinx	The control group tended to have numerically higher medians of AMH, AFC, and number of oocytes retrieved than cases with bilateral or unilateral salpingectomy
Orvieto et al., 2011 [20]	Retrospective study	15	Effect of salpingectomy on ipsilateral ovarian reserve	Reduced ovarian response of the ipsilateral ovary after unilateral salpingectomy
Tavana et al., 2021 [21]	Cohort study	66	TAH + bilateral salpingectomy and TLH	The serum levels of AMH decreased significantly after both methods of hysterectomy (laparoscopy and laparotomy), while this decrease was greater in TAH group

**Table 7:** The effect of prophylactic salpingectomy on ovarian reserve

Author	Study type	Sample size	Type of surgery	Conclusion
Venturella et al., 2017 [22]	Observational study	79	Prophylactic salpingectomy along with total laparoscopic hysterectomy	The safety of the salpingectomy procedure for ovarian reserve was proved
Wang et al., 2021 [23]	Retrospective study	373	Prophylactic salpingectomy along with total laparoscopic hysterectomy	Prophylactic bilateral salpingectomy did not damage the ovarian reserve of reproductive-age women who underwent laparoscopic hysterectomy
Gelderblom et al., 2022 [24]	Systematic review and meta-analysis	Meta-analysis, 1047 studies	Opportunistic salpingectomy	Opportunistic salpingectomy does not result in a significant reduction of ovarian reserve in the short term

**Table 8:** The effect of salpingectomy on IVF PTO: proximal tubal occlusion, IVF-ET: in vitro fertilization and embryo transfer, AFC: antral follicle count

Author	Study type	Sample size	Type of surgery	Conclusion
Vignarajan et al., 2019 [25]	Randomized control trial	165 patients, PTO group 83, salpingectomy group 82	PTO or salpingectomy in patients with hydrosalpinx undergoing IVF-ET	PTO is superior to salpingectomy for patients with hydrosalpinx undergoing IVF-ET in terms of ovarian reserve however, pregnancy rates were comparable
Chen et al., 2020 [26]	Retrospective study	1992 patients, salpingectomy group 534, control group 1388	Salpingectomy versus control on ovarian reserve and IVF outcome	Salpingectomy may decrease AFC but not live birth rate for IVF-ET patients aged 35-39 years
Ho et al., 2022 [27]	Case-control study	54 salpingectomy patients versus 59 without tubal disease	Salpingectomy affecting IVF outcome versus control	A negative effect on the number of retrieved oocytes in the subsequent IVF cycle after salpingectomy is more likely in women aged <35 years with suboptimal ovarian reserve

**Table 9:** The effect of salpingectomy for ectopic pregnancy on ovarian reserve

Author	Study type	Sample size	Type of study	Conclusion
Rodgers et al., 2020 [28]	Prospective study	58 women in the ectopic group and 12 in the miscarriage group	Laparoscopic salpingectomy for ectopic pregnancy on ovarian reserve	Laparoscopic salpingectomy using electrosurgery and mechanical scissors does not damage ovarian reserve
Luo et al., 2019 [29]	Systematic review and meta-analysis	243 articles from the databases and 7 studies were included in the meta-analysis	Effect of salpingectomy for ectopic pregnancy on ovarian reserve	Salpingectomy has no negative effect on the ovarian reserve and ovarian response

In the scenario of an unsettled debate, our study aimed to identify the surgical technique that would minimize the compromise, if any, caused to the ovaries secondary to salpingectomy. In our study, we employed AMH, serum FSH, AFC, and ovarian size pre- and post-salpingectomy as markers for ovarian function. We observed a rise in the mean value of early follicular phase FSH and a fall in AMH postoperatively compared to pre-salpingectomy values in both bipolar electrocautery and ultrasonic shear groups, but this was not statistically significant. These findings suggest that the salpingectomy procedure does cause a minor degree of insult to ovarian function, although not significant. Interruption of collaterals to the ovaries and lateral thermal damage from the energy sources may explain this slight decline in ovarian function. AFC, on the other hand, showed a statistically significant decline in the bipolar electrocautery group compared to the ultrasonic shear group measured during the three-month postoperative period (p=0.0002). The ultrasonic shear, being equipment based on high-frequency ultrasonic energy, has been proven to cause lesser lateral dissipation of energy and, thereby, a lesser degree of damage to non-target organs when compared to its electrosurgical counterparts like electrocautery [13]. This property makes ultrasonic shear a safer alternative, as indicated by the findings in our study. Ovarian size, which was the fourth parameter compared, remained similar on follow-up analysis in both groups and did not show any significance.

The limitations of our study include the limited sample size and short-term follow-up. Future large-scale trials with longer periods of follow-up may bring more clarity to the superior safety margin of ultrasonic shear in salpingectomy.

## Conclusions

Ovarian tissue compromise post-salpingectomy becomes a serious concern, particularly in women in the early reproductive age group with fertility concerns. Our study has shown that salpingectomy using ultrasonic shear causes lesser insult to the residual ovarian reserve, as reflected by postoperative AFC and AMH reserve, in comparison to the standard bipolar electrocautery. The ultrasonic shear technique for salpingectomy is technically an easier and safer alternative to the traditional techniques.

## References

[1] Qin F, Du DF, Li XL (2016). The effect of salpingectomy on ovarian reserve and ovarian function. Obstet Gynecol Surv.

[2] (2019). ACOG committee opinion no. 774: Opportunistic salpingectomy as a strategy for epithelial ovarian cancer prevention. Obstet Gynecol.

[3] Callahan MJ, Crum CP, Medeiros F (2007). Primary fallopian tube malignancies in BRCA-positive women undergoing surgery for ovarian cancer risk reduction. J Clin Oncol.

[4] Arvizo C, Uy-Kroh MJ (2017). Bilateral salpingectomy using percutaneous minilaparoscopy. J Minim Invasive Gynecol.

[5] Yoon SH, Kim SN, Shim SH, Kang SB, Lee SJ (2016). Bilateral salpingectomy can reduce the risk of ovarian cancer in the general population: a meta-analysis. Eur J Cancer.

[6] Findley AD, Siedhoff MT, Hobbs KA, Steege JF, Carey ET, McCall CA, Steiner AZ (2013). Short-term effects of salpingectomy during laparoscopic hysterectomy on ovarian reserve: a pilot randomized controlled trial. Fertil Steril.

[7] Kotlyar A, Gingold J, Shue S, Falcone T (2017). The effect of salpingectomy on ovarian function. J Minim Invasive Gynecol.

[8] Mohamed AA, Yosef AH, James C, Al-Hussaini TK, Bedaiwy MA, Amer SA (2017). Ovarian reserve after salpingectomy: a systematic review and meta-analysis. Acta Obstet Gynecol Scand.

[9] Sezik M, Ozkaya O, Demir F, Sezik HT, Kaya H (2007). Total salpingectomy during abdominal hysterectomy: effects on ovarian reserve and ovarian stromal blood flow. J Obstet Gynaecol Res.

[10] Morelli M, Venturella R, Mocciaro R (2013). Prophylactic salpingectomy in premenopausal low-risk women for ovarian cancer: primum non nocere. Gynecol Oncol.

[11] Gupta V, Agarwal S, Chaudhari P, Saxena N, Nimonkar S (2023). A study to evaluate the effect of opportunistic salpingectomy on ovarian reserve and function. J Obstet Gynaecol India.

[12] Ye XP, Yang YZ, Sun XX (2015). A retrospective analysis of the effect of salpingectomy on serum antiMüllerian hormone level and ovarian reserve. Am J Obstet Gynecol.

[13] Kobayashi M, Kitahara Y, Hasegawa Y, Tsukui Y, Hiraishi H, Iwase A (2022). Effect of salpingectomy on ovarian reserve: a systematic review and meta-analysis. J Obstet Gynaecol Res.

[14] Grynnerup AG, Lindhard A, Sørensen S (2013). Anti-Müllerian hormone levels in salpingectomized compared with nonsalpingectomized women with tubal factor infertility and women with unexplained infertility. Acta Obstet Gynecol Scand.

[15] Yuan Z, Cao D, Bi X, Yu M, Yang J, Shen K (2019). The effects of hysterectomy with bilateral salpingectomy on ovarian reserve. Int J Gynaecol Obstet.

[16] Wu S, Zhang Q, Li Y (2020). Effect comparison of salpingectomy versus proximal tubal occlusion on ovarian reserve: a meta-analysis. Medicine (Baltimore).

[17] Atilgan R, Pala Ş, Kuloğlu T, Şanli C, Yavuzkir Ş, Özkan ZS (2020). Comparison of the efficacy between bilateral proximal tubal occlusion and total salpingectomy on ovarian reserve and the cholinergic system: an experimental study. Turk J Med Sci.

[18] Gelbaya TA, Nardo LG, Fitzgerald CT, Horne G, Brison DR, Lieberman BA (2006). Ovarian response to gonadotropins after laparoscopic salpingectomy or the division of fallopian tubes for hydrosalpinges. Fertil Steril.

[19] Ni L, Sadiq S, Mao Y, Cui Y, Wang W, Liu J (2013). Influence of various tubal surgeries to serum antimullerian hormone level and outcome of the subsequent IVF-ET treatment. Gynecol Endocrinol.

[20] Orvieto R, Saar-Ryss B, Morgante G, Gemer O, Anteby EY, Meltcer S (2011). Does salpingectomy affect the ipsilateral ovarian response to gonadotropin during in vitro fertilization-embryo transfer cycles?. Fertil Steril.

[21] Tavana Z, Askary E, Poordast T, Soltani M, Vaziri F (2021). Does laparoscopic hysterectomy + bilateral salpingectomy decrease the ovarian reserve more than total abdominal hysterectomy? A cohort study, measuring anti-Müllerian hormone before and after surgery. BMC Womens Health.

[22] Venturella R, Lico D, Borelli M (2017). 3 to 5 years later: long-term effects of prophylactic bilateral salpingectomy on ovarian function. J Minim Invasive Gynecol.

[23] Wang S, Gu J (2021). The effect of prophylactic bilateral salpingectomy on ovarian reserve in patients who underwent laparoscopic hysterectomy. J Ovarian Res.

[24] Gelderblom ME, IntHout J, Dagovic L, Hermens RP, Piek JM, de Hullu JA (2022). The effect of opportunistic salpingectomy for primary prevention of ovarian cancer on ovarian reserve: a systematic review and meta-analysis. Maturitas.

[25] Vignarajan CP, Malhotra N, Singh N (2019). Ovarian reserve and assisted reproductive technique outcomes after laparoscopic proximal tubal occlusion or salpingectomy in women with hydrosalpinx undergoing in vitro fertilization: a randomized controlled trial. J Minim Invasive Gynecol.

[26] Chen T, Zhao F, Wang Q (2020). Salpingectomy may decrease antral follicle count but not live birth rate for IVF-ET patients aged 35-39 years: a retrospective study. J Ovarian Res.

[27] Ho CY, Chang YY, Lin YH, Chen MJ (2022). Prior salpingectomy impairs the retrieved oocyte number in in vitro fertilization cycles of women under 35 years old without optimal ovarian reserve. PLoS One.

[28] Rodgers R, Carter J, Reid G, Krishnan S, Ludlow J, Cooper M, Abbott J (2020). The effect of laparoscopic salpingectomy for ectopic pregnancy on ovarian reserve. Aust N Z J Obstet Gynaecol.

[29] Luo J, Shi Y, Liu D (2019). The effect of salpingectomy on the ovarian reserve and ovarian response in ectopic pregnancy: a systematic review and meta-analysis. Medicine (Baltimore).

